# The pregnancy-associated spontaneous coronary artery dissection in a young woman with a novel missense mutation in *NOTCH1*: a case report

**DOI:** 10.1186/s12881-020-01058-2

**Published:** 2020-06-01

**Authors:** Bo Bai, Meng Zhang, Yihao Zhuang, Jirong Zhu, Wenjing Li, Wei Ma, Haibo Chen

**Affiliations:** Department of Cardiology, Shenzhen Second People’s Hospital, the First Affiliated Hospital of Shenzhen University Health Science Center, No. 3002, Sungang West Road, Futian District, Shenzhen, 518035 China

**Keywords:** Spontaneous coronary artery dissection, *NOTCH 1* variant, Myocardial infarction, Primary percutaneous coronary intervention, Pregnancy

## Abstract

**Background:**

Spontaneous coronary artery dissection (SCAD) is frequently reported as a disorder that primarily affects women without risk factors for cardiovascular disease. Although it has been recognized as one of the genetically mediated vascular disorders, the genetic pathogenesis of SCAD remains obscure to date.

**Case presentation:**

In this report, we presented a rare case of pregnancy-associated SCAD in a young woman that occurred in multiple coronary arteries within a short period. The initial conservative management and then intravascular ultrasound-guided primary percutaneous coronary intervention (PCI) were adopted to achieve optimal results of revascularization in affected coronary arteries and avoid potential risks for PCI-associated complications. We further performed the whole-exome sequencing and Sanger sequencing and, for the first time, reported a novel heterozygous missense variant, c.4574 C > T (p.Arg1438Cys), in the *NOTCH1* gene. This variant has never been documented in the medical literature and was predicted as being potentially damaging or disease-causing variant.

**Conclusions:**

We described a rare case of recurrent SCAD in a young woman after baby delivery. The initial conservative management and PCI with multiple stent implantations were successfully implemented to achieve optimal results of revascularization in coronary arteries. We, for the first time, identified a novel missense variant in the *NOTCH1* gene, which appears to be a potential predisposing factor for artery fragility.

## Background

Spontaneous coronary artery dissection (SCAD) is defined as an epicardial coronary artery dissection that is not associated with atherosclerosis or trauma and not iatrogenic [1]. It has been deemed rare historically and frequently reported in young women with pregnancy or within the peripartum period [2, 3]. Despite a relatively low incidence of pregnancy-associated SCAD, pregnant women with SCAD seem to have more acute presentations and poorer prognosis, compared to women with SCAD unrelated to pregnancy [2–4]. The majority of patients experienced acute ST-elevation myocardial infarction (STEMI), cardiogenic shock, cardiac arrest, and maternal death [2–4]. However, the precise nature of this association remains to be elucidated. It is likely to be driven by multiple predisposing factors, such as underlying arteriopathies, genetic factors, hormonal influences, inherited or acquired arteriopathies, or yet-undefined mechanisms [1, 5, 6].

The cohort studies and case reports have provided suggestive findings that SCAD is one of the genetically mediated vascular disorders. Mutation in many genes that are related to arterial fragility and dissections have been reported in SCAD cases, including variations in *TSR1, FBN1, ACTA2, SMAD3, COL3A1, TGFBR1/R2* [6–9]. Due to insufficient cases discovered, the genetics of SCAD remains obscure to date. Identification of potential risk loci will broaden our knowledge of SCAD pathogenesis as well as diagnosis, treatment, and prognosis.

## Case presentation

**Fig. 1:**
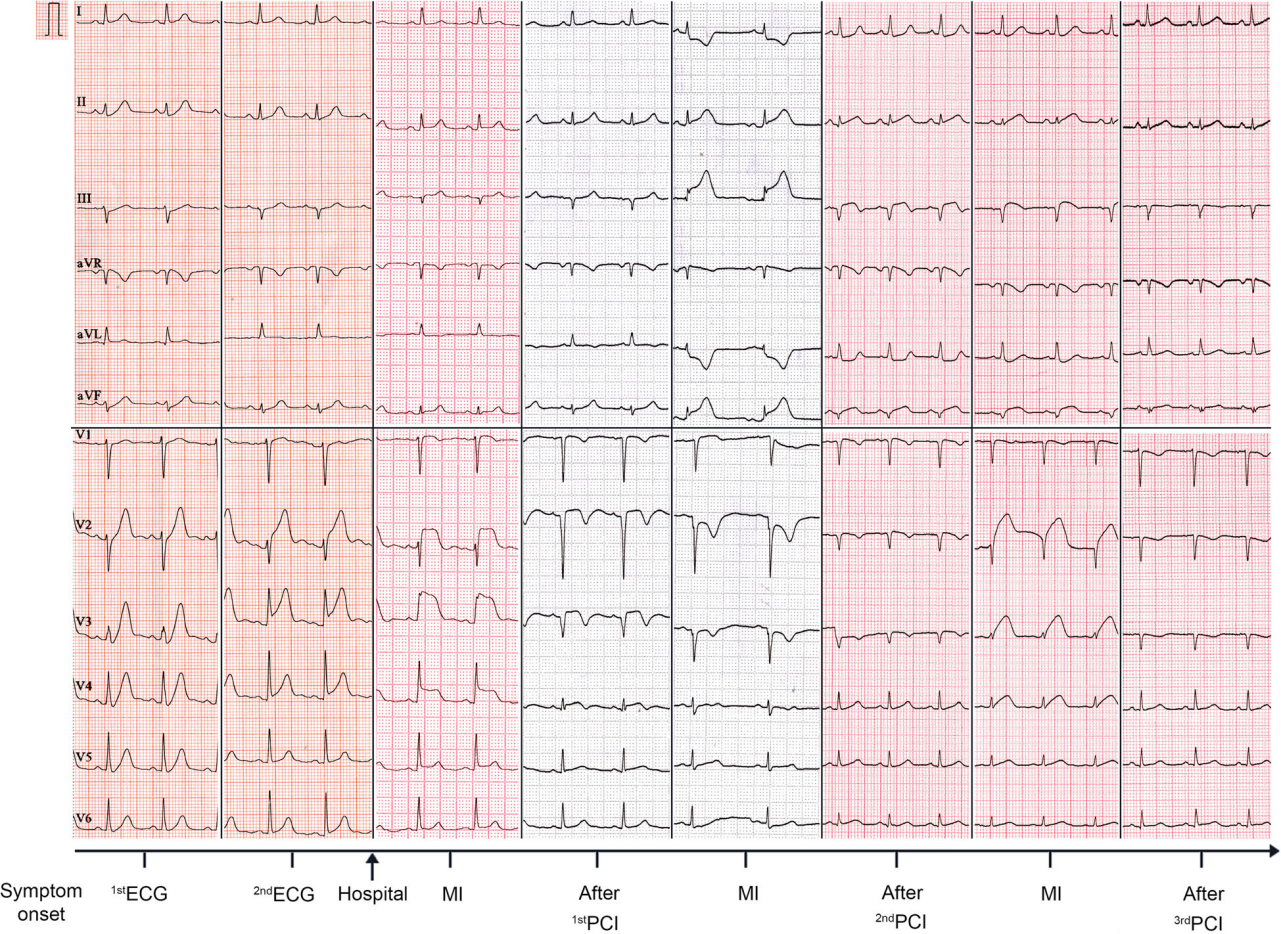
The dynamic changes in the electrocardiogram (ECG) of a patient from symptom onset to discharge of the hospital. PCI: percutaneous coronary intervention, myocardial infarction (MI)

**Fig. 2:**
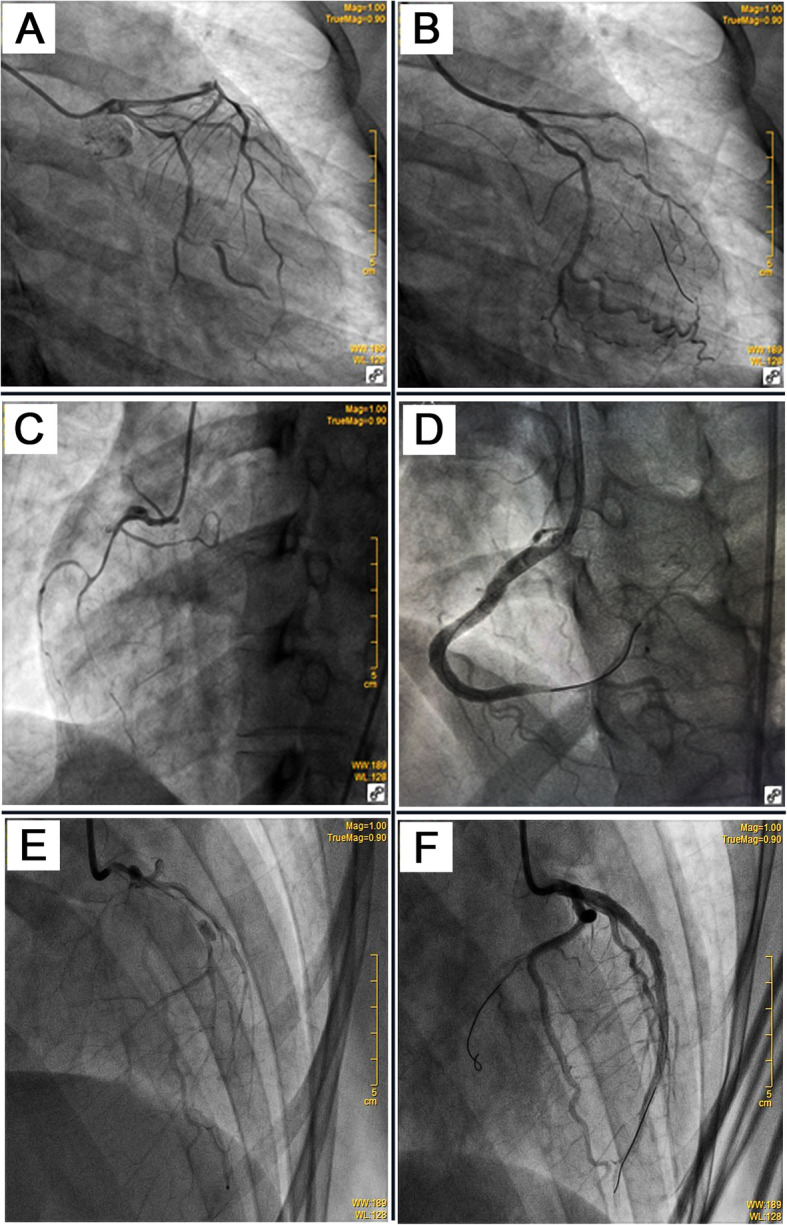
**a** Left coronary angiogram showed long diffuse stenosis in LAD and LCX. **b** The TIMI 3 flow was restored in distal LAD after dilatation with undersized balloons at low-pressure. **c** Three days after the initial PCI, the MI recurred in this patient. The angiography showed coronary dissection in the ostium of the right coronary artery with TIMI1 flow 1. **d** No residual stenosis was achieved after implantation with multiple drug-eluting stents from distal to proximal segments of the right coronary artery. **e** Three days later, the patient suffered from MI again. Coronary angiogram demonstrated long diffuse stenosis in LAD and LCX with TIMI flow 3. **f** Multiple drug-eluting stents were implanted into focal lesions of LAD, LCX, and LMC arteries to seal intimal tears and achieve the optimal results of no residual stenosis and restoration of TIMI 3 flow. LAD: left anterior descending coronary artery; LCX: left circumflex coronary artery; LMC: left main coronary artery; TIMI: Thrombolysis in Myocardial Infarction

**Fig. 3:**
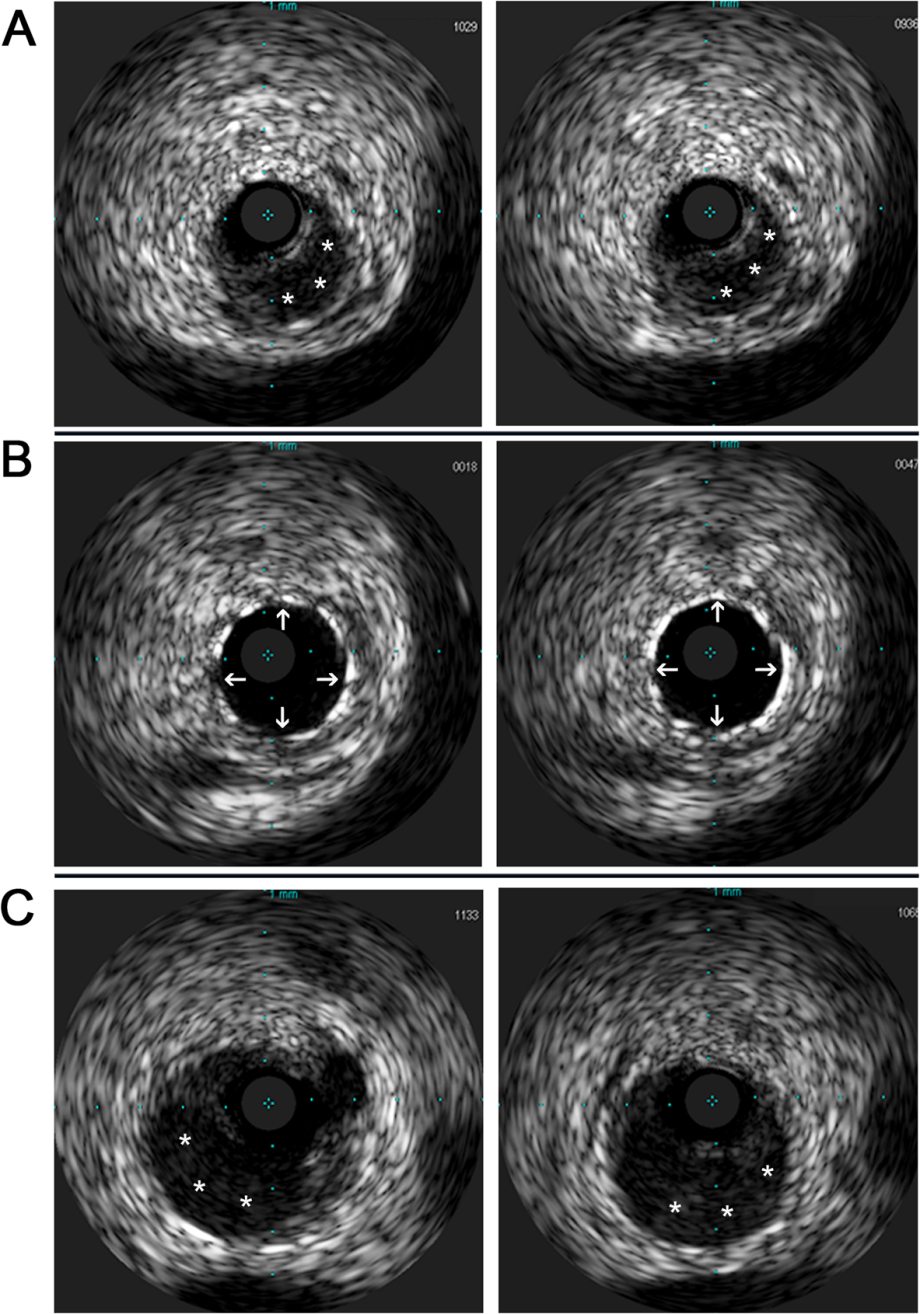
**a** Intravascular ultrasound (IVUS) images were presented to show the artery dissection starting from the proximal right coronary artery and ascertained the guidewire passed through the true lumen properly (left and right). **b** IVUS demonstrated stents attached to endothelium well and covered artery dissections completely (left and right). **c** IVUS showed multiple focal artery dissections located at LMC, LAD, as well as the ostium and the proximal segment of LCX (left and right). The intramural hematoma was demonstrated (*), and implanted stents were indicated with arrows

A 30-year-old woman suffered from sustained chest pain in the early postpartum period (15 h after vaginal delivery). She had no history of hypertension, diabetes, and smoking. The electrocardiogram (ECG) demonstrated the upsloping ST-segmental depression in precordial leads and positive symmetric T waves, indicating the de Winter syndrome, a condition associated with acute occlusion of the left anterior descending coronary artery (LAD) (Fig. 1) [10]. Twenty minutes later, the ECG showed the typical ST-segment elevation in leads V1 to V4 (Fig. 1). She was administrated with 300 mg aspirin and immediately transferred to our hospital. The repeated ECG indicated the tombstoning ST-segment elevation in leads V2 and V3 (Fig. 1) [11]. The laboratory results showed hs-troponin I of 0.46 ng/mL (normal range < 0.02 ng/mL) and CK-MB of 24 ng/mL (normal range < 7.2 ng/mL), supporting a diagnosis of the STEMI. Echocardiography demonstrated unsynchronized left ventricular wall motion but normal systolic function (EF = 64%). After administration with 180 mg ticagrelor, the timely primary percutaneous coronary intervention (PCI) was implemented to this patient. Coronary angiogram revealed the presence of long diffuse stenosis from ostium to distal end of LAD with Thrombolysis in Myocardial Infarction (TIMI) flow 3. We noticed the contrast hold-up near to mid-segments of LAD and considered which was likely attributable to SCAD, coronary vasculitis, or intramural hematoma (IMH) (Fig. 2a). A Run-through NS Extra Floppy guidewire was used to cross LAD to the distal end. Sprinter balloon was then positioned at proximal-middle LAD, and undersized balloons at low-pressure dilatation were performed to achieve a good angiographic result (Fig. 2b). After PCI, she did not complain about any discomfort, and the chest pain was substantially relieved. The ECG showed sinus rhythm and the disappearance of ST-segment elevation (Fig. 1). The dual-antiplatelet and antithrombotic therapies were then carried on by administration of aspirin (100 mg/day), clopidogrel (75 mg/day), and subcutaneous low molecular weight heparin (0.4 mL, twice per day). During the following 2 days, she had no chest discomfort. Three days after the initial PCI, she re-experienced intense chest pain and sweating. The ECG indicated ST-segment elevation in leads II, III, and AVF (Fig. 1), indicating acute MI in the inferior wall and right ventricle. After administration with 300 mg aspirin and 300 mg clopidogrel, she underwent the PCI immediately. Coronary angiogram demonstrated the SCAD in the ostium of the right coronary artery (RCA) with TIMI flow 1 (Fig. 2c). A Run-through NS Extra Floppy guidewire was used to cross RCA to the distal end. The intravascular ultrasound (IVUS) ascertained the guidewire passed through the true lumen properly and showed the artery dissection starting from proximal RCA. The IMH in the mid-to-distal segments compressed the true lumen (Fig. 3a, left and right). After pre-dilations by Sprinter balloon at proximal-to-mid RCA, the multiple drug-eluting stents were implanted sequentially from distal to proximal segments and the ostium of RCA to restore TIMI flow 3 with no residual stenosis (Fig. 2d). IVUS showed stents attached to endothelium well and covered artery dissections completely (Fig. 3b, left and right). After PCI, the patient did not complain about chest pain and had a normal cardiac function (EF = 64%). The ECG indicated the elevation of ST-segment disappeared in multiple leads (Fig. 1). However, 3 days after the second PCI, she suffered from MI again (Fig. 1). Coronary angiogram demonstrated long diffuse stenosis in LAD and LCX with TIMI flow 3 (Fig. 2e). IVUS showed that multiple artery dissections located in mid-to-distal segments of the left main coronary artery (LMC), LAD, as well as the ostium and proximal segment of LCX (Fig. 3c, left and right). We decided to intervene in the LMC, LAD, and LCX arteries by stent implantation. Multiple drug-eluting stents were positioned and implanted into focal lesions of LAD, LCX, and LMC arteries to seal intimal tears. The optimal results of no residual stenosis and restoration of TIMI 3 flow in coronary arteries were achieved (Fig. 2f). During the following week, antiplatelet and antithrombotic therapies were continued for this patient. She had no more chest pain. The hs-troponin I progressively reduced to 0.24 ng/mL. The echocardiography showed slightly decreased systolic function (EF = 57%), normal diastolic function, but unsynchronized ventricular wall motion. She recovered well and then was discharged home. One month after discharge, follow-up at the outpatient department showed she had no chest discomfort.

### Genomic analysis

**Fig. 4:**
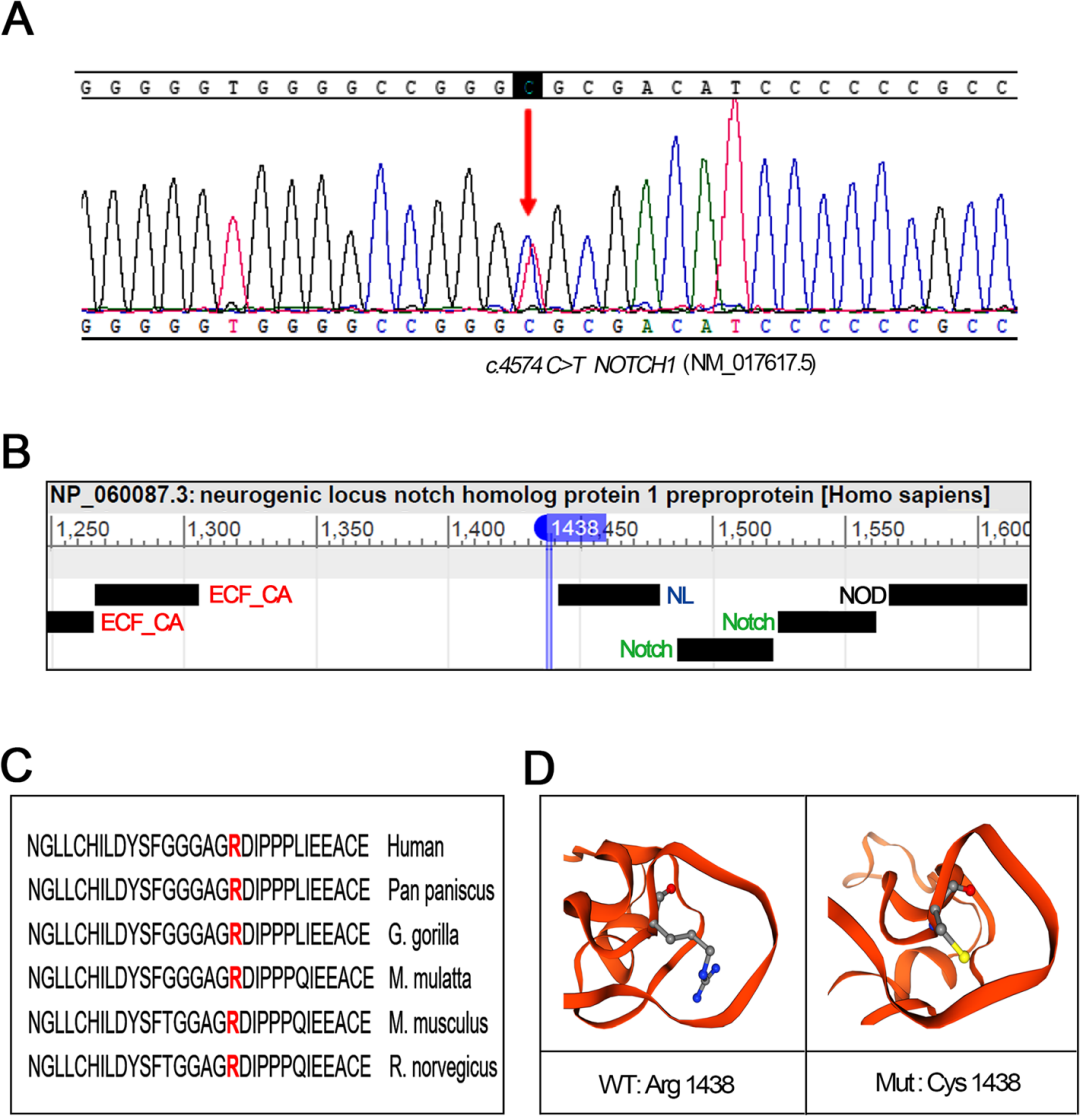
**a** Sanger sequencing confirmed that the patient was heterozygous for *NOTCH1* c.4574 C > T (p.Arg1438Cys) variant. **b** A schematic diagram of the domain regions of neurogenic locus notch homolog protein 1 preproprotein (NP_060087.3, amino acid:1250 to 1600). The mutant residue was located very close to the NL domain. EGF_CA: Calcium-binding EGF-like domain; NL: Domain found in Notch and Lin-12; Notch: Lin-12/Notch repeat; NOD: NOTCH domain. **c** The protein sequence alignment showed the variant region was conserved among species. **d** The structure modeling of wildtype (WT) and mutant (Mut) Notch1 was built using SWISSMODEL

Her father died at the age of 55 years due to MI. Her paternal aunt also suffered from MI and died 3 days after child delivery. It does appear that a heritable disorder was present in her family. In order to find potentially causative gene variants, we performed the whole-exome sequencing (WES) using genomic DNA isolated from the peripheral blood of this patient. The WES was done on MGISEQ-200 Sequencer (MGI Tech Co., Limited, China). The WES achieved an average coverage of≥100 X on target and 95% of bases covered at≥20X. We screened for rare variants with a MAF of < 0.01 (PM2: Extremely low frequency, ACMG guideline) based on the databases of the 1000 Genomes project and ESP 6500. We further validated variants by Sanger sequencing. Sequence alternations were reported according to Human Genome Variation Society guidelines (HGVS) and mapped to Human Genome Build GRCh37/UCSC hg19(https://genome.ucsc.edu/cgi-bin/hgGateway?db=hg19). It is noteworthy that no well-known pathological variants (e.g., *TSR1, FBN1, ACTA2, SMAD3, COL3A1, TGFBR1/R2*) were found in this patient, whereas we identified a novel heterozygous missense variant, c.4574 C > T (p.Arg1438Cys), in the *NOTCH1* gene (https://www.ncbi.nlm.nih.gov/gene/?term=NM_017617.5) (Fig. 4a). Importantly, this missense mutation was not found in the non-SCAD cohort controls of China [8]. It is a quite rare variant with the dbSNP allele frequency of 9.023e^− 6^. The residue is located very close to the NL domain (Domain found in Notch and Lin-12, amino acid:1442–1479) of neurogenic locus notch homolog protein 1 preproprotein (https://www.ncbi.nlm.nih.gov/protein/NP_060087.3) (Fig. 4b). It is a conserved residue among species, including human, chimpanzee, monkey, mouse, and rat (Fig. 4c). The structure models of wildtype (WT) and mutant (Mut) protein were built using SWISSMODEL (https://www.swissmodel.expasy.org). It appears that the substituted cysteine did not apparently change the senior structure of the variant protein (Fig. 4d). Then, the in-silico software of the computational algorithms SIFT (Sorting Intolerant from Tolerant, http://sift.jcvi.org), PolyPhen2 (Polymorphism Phenotyping, http://genetics.bwh.harvard.edu/pph.harvard.edu/pph22) and Mutation Taster (http://www.mutationtaster.org) were used to predict the pathogenicity of this novel variant. While SIFT suggested it as being a tolerated variant, PolyPhen2 and Mutation Taster prediction tools presented it as being potentially damaging or disease-causing variant, respectively (PP3: Multiple lines of computational evidence support a deleterious effect on the gene or gene product, ACMG guideline).

## Discussion and conclusions

In terms of therapeutic strategies for SCAD, conservative management and PCI have been well acknowledged. However, the decisions should be individualized and contemplated in the context of the coronary anatomy, the stability of vessel dissection, ongoing ischemia, and hemodynamic conditions [5, 6]. Considering the patient had the hemodynamic stability with TIMI3 flow in the culprit artery, we initially preferred the minimalist mechanical intervention by undersized balloons at low-pressure dilatation. We did not continue with a more revascularization strategy to allow the chance of “healing” of suspicious SCAD lesions to occur. Unfortunately, this case is quite rare because the SCAD with MI reoccurred within a short period. Therefore we adopted optimized PCI approaches to improve the outcomes of revascularization and avoid potential risks for PCI-associated complications [12, 13]. First, we performed coronary angiography with femoral access to reduce the risk of catheter-induced iatrogenic dissection. Owing to the complex upper limb vessel anatomy, the catheter-induced injuries were frequently reported in SCAD patients when radial access was used [14]. Second, we used IVUS to aid the diagnosis of SCAD, which was also helpful for us to ascertain the guidewire passed through the true lumen properly, a critical step when revascularization procedures were pursued. Third, the multiple drug-eluting stents were sequentially implanted to target and seal focal dissections of coronary arteries. The patient had successful procedural outcomes.

This patient showed a susceptive family history of an inherited systematic arteriopathy. Therefore, we were motivated to explore the potential genetic variants further. We performed WES and Sanger sequencing on the index patient and, for the first time, reported a novel heterozygous *NOTCH1* variant that has never been documented in the medical literature. *NOTCH1* encodes a large protein containing an extracellular domain with epidermal growth factor (EGF)-like repeats, cysteine-rich Notch/LIN-12 repeats, an intracellular domain with ankyrin repeats, and a transmembrane domain. The Notch signaling pathway is highly conserved across species. Binding of the Notch receptor ligands, Jagged or Delta-like, leads to enzymatic cleavage and nuclear translocation of the Notch intracellular domain (NICD). NICD binds to DNA-binding protein CSL, leading to transcriptional activation of downstream target genes (e.g., *Hes* and *Hey*) and cell fate determination [15]. Notch1 signaling plays a crucial role in vascular smooth muscle cell (VSMC) apoptosis. The defective Notch1 signaling potentiates the changes in VSMC apoptosis and proliferation [16]. VSMC-specific Notch-1 heterozygous deficient mice showed decreased VSMC proliferation but increased cell apoptosis [17]. Notch1 is also critical to coordinate VSMC-endothelial cell contact and maintain monolayer integrity and vascular homeostasis in response to injury [18]. Mutations in *NOTCH1* (e.g., p.Thr596Met, p.Arg1108X, p.Ala1343Val, p.Pro1390Thr, p.His1505 del, p.Pro1797His) are implicated in bicuspid aortic valve aortopathy, a condition associated with abnormal apoptosis and differentiation of VSMCs [19–21]. Yet, a pathophysiological association between *NOTCH1* mutation and artery dissections has not been well established. A case study reported that patients carrying *NOTCH1* mutation (p.Pro2122Leu) showed spontaneous and recurrent dissections of extracranial arteries [22]. A recent Chinese SCAD cohort study identified 27 *NOTCH1 * variants, but most of them also occur in non-SCAD controls. There are two variants specifically identified in SCAD cases. The prediction tools presented them as being tolerated (p.Asp1277Asn) and damaging (p.Glu1143Gly), respectively [8]. Of note, the p.Arg1438Cys variant was neither found in these non-SCAD controls. Although this variant has an extremely low frequency (MAF < 0.01), we are not able to validate this variant in her mother to provide PS2 evidence and further conclude it as a likely pathogenic variant (ACMG guideline). This variant is classified as disease-causing by in silico prediction. However, structural modeling did not find an apparent change in the senior structure. Therefore, the evidence to conclude its potential pathogenicity remains insufficient. We are currently examining the ligand binding and Notch1-dependent differentiation of VSMC after transfection with variant. It is of significant interest to investigate the pathophysiological association between this missense mutation and vessel fragility via more detailed experiments.

In conclusion, we describe a rare case of recurrent SCAD in a young woman after baby delivery. The initial conservative management and then IVUS-guided PCI achieved optimal results of revascularization in affected coronary arteries. We, for the first time, identified a novel missense variant in the *NOTCH1* gene, which appears to be a potential predisposing factor for artery fragility.

## Data Availability

The data used and analyzed in the present report were deposited in the NCBI Sequence Read Archive (SRA) database. The data are accessible via the SRA accession: PRJNA610829.

## Abbreviations

ECG: Electrocardiogram
EGF: Epidermal growth factor
IMH: Intramural hematoma
IVUS: Intravascular ultrasound
LAD: Left anterior descending coronary artery
LCX: Left circumflex artery
LMC: Left main coronary artery
MI: Myocardial infarction
PCI: Primary percutaneous coronary intervention
RCA: Right coronary artery
SCAD: Spontaneous coronary artery dissection
STEMI: ST-elevation myocardial infarction
TIMI: Thrombolysis in myocardial infarction
VSMC: Vascular smooth muscle cell
WES: Whole-exome sequencing
